# Acquired resistance to first-line immune checkpoint inhibitors in esophageal squamous cell carcinoma: progression patterns and prognostic implications

**DOI:** 10.3389/fimmu.2026.1906395

**Published:** 2026-07-23

**Authors:** Rui-Ying Zhang, Han Wu, Pei-Xiang Hao, Lu-Lu Guan, Dao Xin, Feng Wang

**Affiliations:** 1 Department of Oncology, The First Affiliated Hospital of Zhengzhou University, Zhengzhou, China; 2 Tianjian Laboratory for Advanced Biomedical Sciences, Zhengzhou, China; 3 State Key Laboratory of Metabolic Dysregulation & the Prevention and Treatment of Esophageal Cancer, Zhengzhou, China

**Keywords:** acquired resistance, esophageal squamous cell carcinoma, immune checkpoint inhibitor, oligoprogression, overall survival, progression pattern

## Abstract

**Background:**

Despite the survival benefit of first-line immune checkpoint inhibitor (ICI)-based therapy in advanced esophageal squamous cell carcinoma (ESCC), acquired resistance (AR) remains a major clinical challenge, and its progression patterns and prognostic significance are poorly defined. This study aimed to characterize progression patterns and sites at AR after first-line ICI-based therapy in ESCC and to evaluate their associations with clinical outcomes.

**Methods:**

We retrospectively identified patients with metastatic or unresectable locally advanced ESCC who underwent first-line ICI-based therapy at the First Affiliated Hospital of Zhengzhou University and Anyang Cancer Hospital (September 2023–January 2026). Initial clinical benefit was defined as complete response, partial response, or stable disease lasting ≥6 months; AR was defined as subsequent disease progression or death. Progression was classified as oligoprogression (≤2 sites) or polymetastatic progression (≥3 sites). Overall survival (OS) was estimated using the Kaplan–Meier method and analyzed with Cox models.

**Results:**

Among 1,537 patients treated with ICIs, 106 patients with ESCC who developed AR were included. Oligoprogression was the most common AR pattern (85/106, 80.2%), whereas 21 (19.8%) had polymetastatic progression. Polymetastatic progression was associated with worse OS than oligoprogression (HR = 1.97, 95% CI 1.12–3.47; p = 0.016).

**Conclusions:**

Oligoprogression was the dominant pattern of AR, and lymph nodes were the most frequent progression sites. Polymetastatic progression was associated with poorer OS.

## Introduction

Esophageal cancer remains a major global health burden, ranking 11th in incidence and 7th in cancer-related mortality worldwide (1). In recent years, phase 3 trials such as CheckMate 648, ESCORT-1st, and RATIONALE-306 have established immune checkpoint inhibitor (ICI) combined with chemotherapy as a standard first-line regimen for advanced esophageal squamous cell carcinoma (ESCC) (2–4). Even with the introduction of ICI-based regimens, long-term prognosis remains unsatisfactory (5, 6).

Even after initial clinical benefit, a considerable number of patients subsequently experience disease progression. Long-term follow-up studies across tumor types provide insight into the frequency of AR. In melanoma, 71% of patients treated with nivolumab in CheckMate 067 had progressed by 5 years, whereas long-term analyses of patients with previously treated non-small cell lung cancer who had achieved complete response or partial response at 6 months showed corresponding progression rates of approximately 62% at 4 years (7, 8). Available evidence suggests that AR rates vary across tumor types (7–9).

Clinical observations from other tumor types suggest that acquired resistance is clinically heterogeneous (9). In NSCLC, Gettinger and colleagues found that AR most often manifested in lymph nodes, and that recurrence involved only one or two sites in 88% of patients (10). In melanoma, site-specific analyses also showed that the size and location of metastases were associated with patterns of progression and outcome after combined checkpoint blockade (11). In gastrointestinal malignancies, a Peking University cohort showed that acquired resistance most often presented as oligoprogression, with lymph nodes representing the most frequent sites of progression, and that progression pattern was associated with overall survival (12).

By contrast, evidence focused specifically on ESCC remains limited. In ESCC, it is still uncertain whether survival after AR varies according to progression pattern or the distribution of involved sites. To address this question, we performed a multicenter retrospective study to characterize progression patterns and anatomical site distribution at AR and to examine their associations with overall survival in patients with metastatic or unresectable locally advanced ESCC who had achieved initial clinical benefit from first-line ICI-based therapy.

## Materials and methods

### Study design and patient selection

A multicenter retrospective study was performed at The First Affiliated Hospital of Zhengzhou University and Anyang Cancer Hospital. We included patients with metastatic or unresectable locally advanced ESCC who had received first-line ICI-based therapy between September 1, 2023, and January 1, 2026. Ethical approval was obtained before study initiation, and all procedures complied with the Declaration of Helsinki (13).

Only patients who achieved initial clinical benefit from ICI-based therapy and subsequently developed acquired resistance (AR) were included in this analysis. Initial clinical benefit included complete response, partial response, and stable disease for at least 6 months. Subsequent progression or death was considered acquired resistance (14). Early progressive disease (early PD) was defined as disease progression confirmed by radiographic or pathological assessment within the first two cycles of first-line ICI-based therapy. During ICI treatment, imaging assessments were reviewed by two experienced investigators, and treatment response was determined according to RECIST version 1.1 (15). Progression sites and progression patterns were recorded at the time of acquired resistance. Any discrepancies in response assessment or progression pattern classification were resolved by consensus. Patients were excluded if the available medical records were insufficient to ascertain key clinicopathologic characteristics, treatment response, AR status, or follow-up outcomes.

### Definitions of AR progression patterns and treatment categories

AR progression patterns were predefined according to the number of involved anatomical sites at the time of acquired resistance, with reference to site-based classifications used in prior studies of acquired resistance to immune checkpoint inhibitors (10, 12, 16). Oligoprogression was defined as progression involving one or two sites, whereas polymetastatic progression was defined as progression involving three or more sites. Nodal disease was coded by anatomical region, with cervical/supraclavicular, mediastinal, and abdominal lymph nodes treated as separate sites; multiple lesions within the same region were counted as a single site. Axillary and inguinal nodal involvement, when present, were recorded separately. Each extranodal organ was counted as one site regardless of the number of lesions. For treatment classification, ICI-based regimens were grouped into chemotherapy-containing and non-chemotherapy-containing categories. Chemotherapy-containing regimens consisted of ICI plus chemotherapy, with or without targeted therapy, whereas non-chemotherapy-containing regimens comprised ICI monotherapy or ICI plus targeted therapy. Detailed regimen distributions are summarized in **Table 1**.

**Table 1:** Baseline characteristics of the acquired resistance cohort (n = 106).

Characteristic	Total (n = 106)
Sex, n (%)	
Male	82 (77.4)
Female	24 (22.6)
Age, n (%)	
<65 years	48 (45.3)
≥65 years	58 (54.7)
Cigarette smoking history, n (%)	
Yes	35 (33.0)
No	71 (67.0)
Alcohol consumption history, n (%)	
Yes	22 (20.8)
No	84 (79.2)
Disease status, n (%)	
Locally advanced (unresectable)	22 (20.8)
Metastatic	84 (79.2)
Primary tumor location, n (%)	
Upper	11 (10.4)
Middle	63 (59.4)
Lower	32 (30.2)
Sites of disease involvement, n (%)	
Lymph nodes	92 (86.8)
Liver	17 (16.0)
Lung	27 (25.5)
Bone	6 (5.7)
Pleura	5 (4.7)
Pancreas	3 (2.8)
Other	5 (4.7)
Previous therapy, n (%)	
Surgery	31 (29.2)
Radiotherapy	8 (7.5)
Regimen, n (%)	
ICI monotherapy	2 (1.9)
ICI + chemotherapy	65 (61.3)
ICI + targeted therapy	14 (13.2)
ICI + chemotherapy + targeted therapy	25 (23.6)

ICI, immune checkpoint inhibitor.

### Outcomes

Overall survival (OS) was the primary endpoint and was defined as the time from initiation of ICI-based therapy to death. Time to acquired resistance (AR) was measured from the first ICI dose to the onset of AR.

### Statistical analysis

Survival distributions were described with Kaplan–Meier analysis and compared across groups by the log-rank test. Associations between clinicopathologic variables and survival were examined using Cox proportional hazards regression in univariable and multivariable analyses, with results expressed as hazard ratios (HRs) and 95% confidence intervals (CIs). Variables considered clinically relevant or associated with OS in univariable analysis were entered into the multivariable model. Median follow-up was summarized for the overall cohort and treatment-regimen subgroups. Key baseline characteristics were also compared between study centers as a descriptive assessment of center-level comparability. Fisher’s exact test was used for categorical variables, and the results are presented in **Supplementary Table 2**. Statistical analyses were conducted in R (version 4.5.0) and GraphPad Prism (version 10.6.0). All tests were two-sided, and p values below 0.05 were considered statistically significant.

## Results

### Patient selection

**Figure 1:**
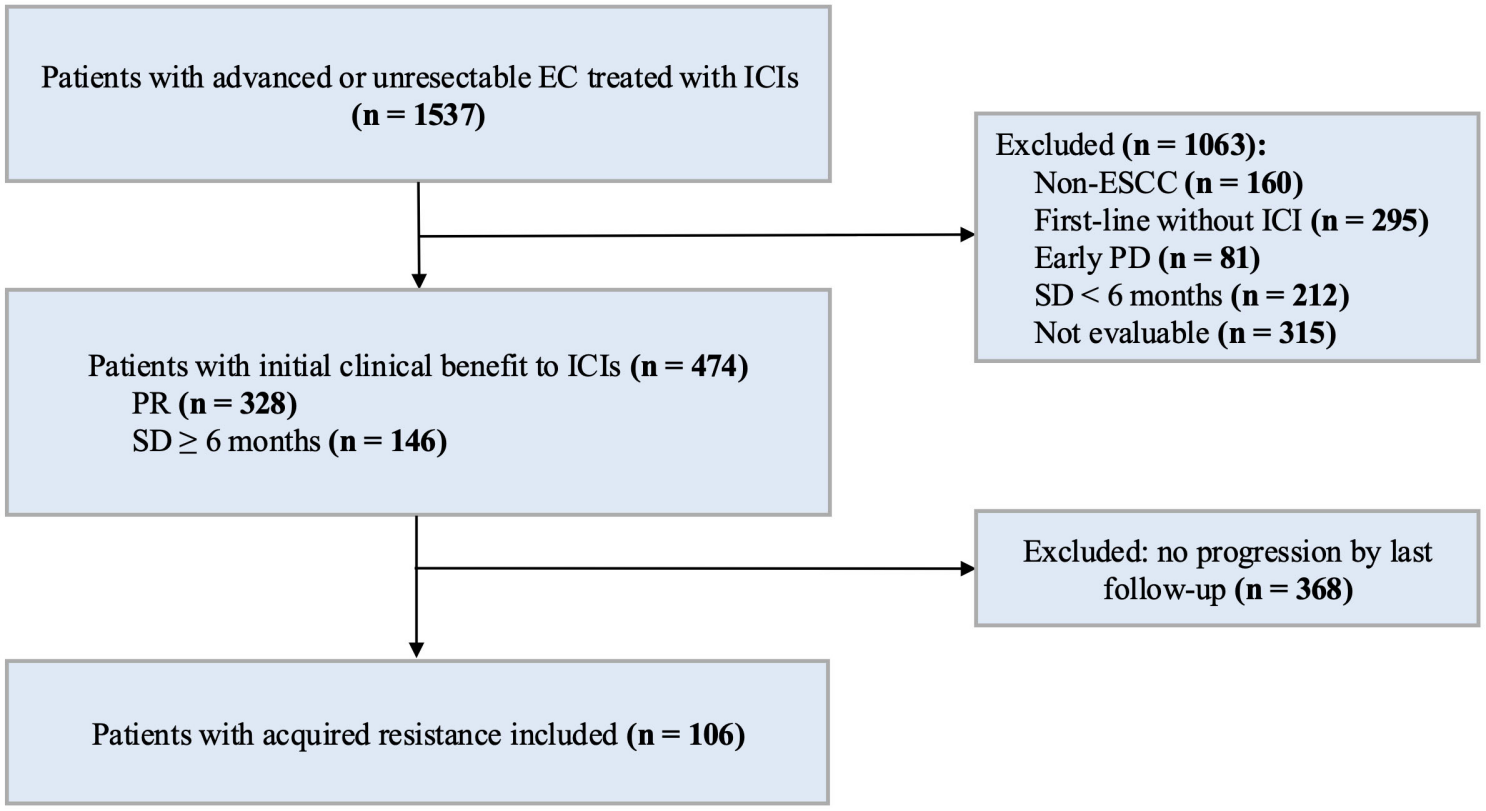
Flow chart of patient selection. AR, acquired resistance; EC, esophageal cancer; ESCC, esophageal squamous cell carcinoma; ICI, immune checkpoint inhibitor; PD, progressive disease; PR, partial response; SD, stable disease.

Among 1,537 ICI-treated patients with metastatic or unresectable locally advanced esophageal cancer, 106 patients with ESCC who developed acquired resistance after initial clinical benefit were included. In the initial screening, 1,063 patients were excluded, mainly because of non-ESCC histology, non–first-line ICI treatment, lack of durable initial clinical benefit, or inadequate evaluability. Among the 474 patients who achieved initial clinical benefit, 328 had partial response and 146 had stable disease maintained for at least 6 months. Of these patients, 368 remained progression-free at the last follow-up and were therefore not included in the AR cohort. Details of patient selection are presented in **Figure 1**.

### Characteristics of patients with acquired resistance

A total of 106 patients with ESCC developed acquired resistance after achieving initial clinical benefit from first-line ICI-based therapy. The cohort was predominantly male (82/106, 77.4%), and most patients had distant metastatic disease at treatment initiation (84/106, 79.2%); the remaining 22 patients (20.8%) had unresectable locoregionally advanced disease. Primary tumors arose most frequently in the middle thoracic esophagus (63/106, 59.4%). At baseline, lymph nodes were the most common disease sites (92/106, 86.8%), followed by the lung (27/106, 25.5%) and liver (17/106, 16.0%). Previous surgery and radiotherapy had been administered in 31 (29.2%) and 8 (7.5%) patients, respectively.

With respect to initial treatment pattern, most patients received chemotherapy-containing ICI-based regimens, including ICI plus chemotherapy (65/106, 61.3%) and ICI combined with chemotherapy and targeted therapy (25/106, 23.6%). The remaining patients were treated with non-chemotherapy-containing regimens, namely ICI monotherapy (2/106, 1.9%) or ICI plus targeted therapy (14/106, 13.2%). The median follow-up was 39.5 months (95% CI, 33.4–51.1) in the overall cohort, 41.3 months (95% CI, 33.7–51.4) in the chemotherapy-containing subgroup, and 33.0 months (95% CI, 22.1–35.5) in the non-chemotherapy-containing subgroup. When key baseline characteristics were compared between The First Affiliated Hospital of Zhengzhou University and Anyang Cancer Hospital, no statistically significant differences were observed in the evaluated variables, including age, sex, disease status, primary tumor location, baseline disease involvement, previous therapy, treatment category, and initial clinical benefit (**Supplementary Table 2**).

**Figure 2:**
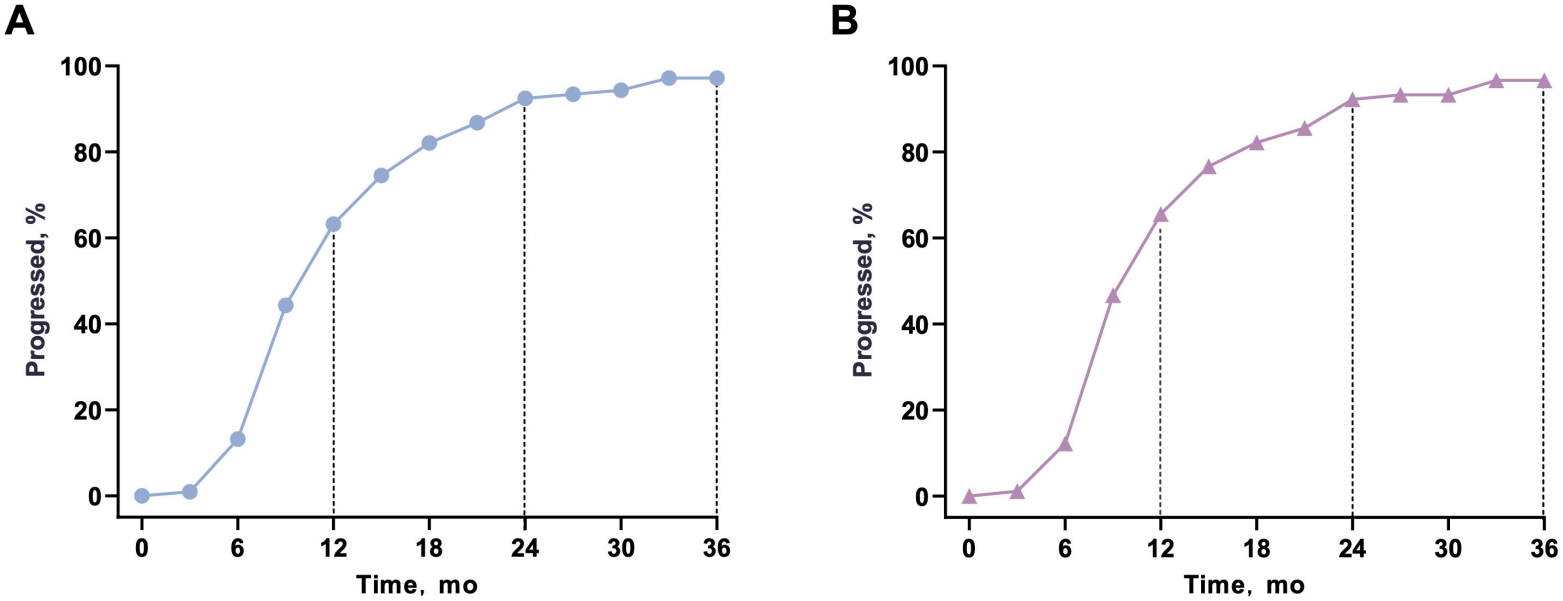
Time to acquired resistance in the overall cohort and the chemotherapy-containing cohort. Panel **(A)** shows the rate of acquired resistance over time in the overall AR cohort. Panel **(B)** shows the rate of acquired resistance over time in the chemotherapy-containing cohort. AR, acquired resistance; mo, months.

In the overall AR cohort, the median OS was 28.6 months (95% CI, 25.3–35.3), and the median interval from treatment initiation to acquired resistance was 9.8 months (95% CI, 8.4–11.2). Most AR events occurred within the first 12 months after treatment initiation, and nearly all had occurred by 2 years (**Figure 2A**). We then examined time to AR among patients treated with chemotherapy-containing regimens. In this cohort, patients had a median OS of 28.0 months (95% CI, 24.6–37.9), and the corresponding median interval to AR was 9.5 months (95% CI, 8.2–11.0) (**Figure 2B**).

### Progression patterns

**Figure 3:**
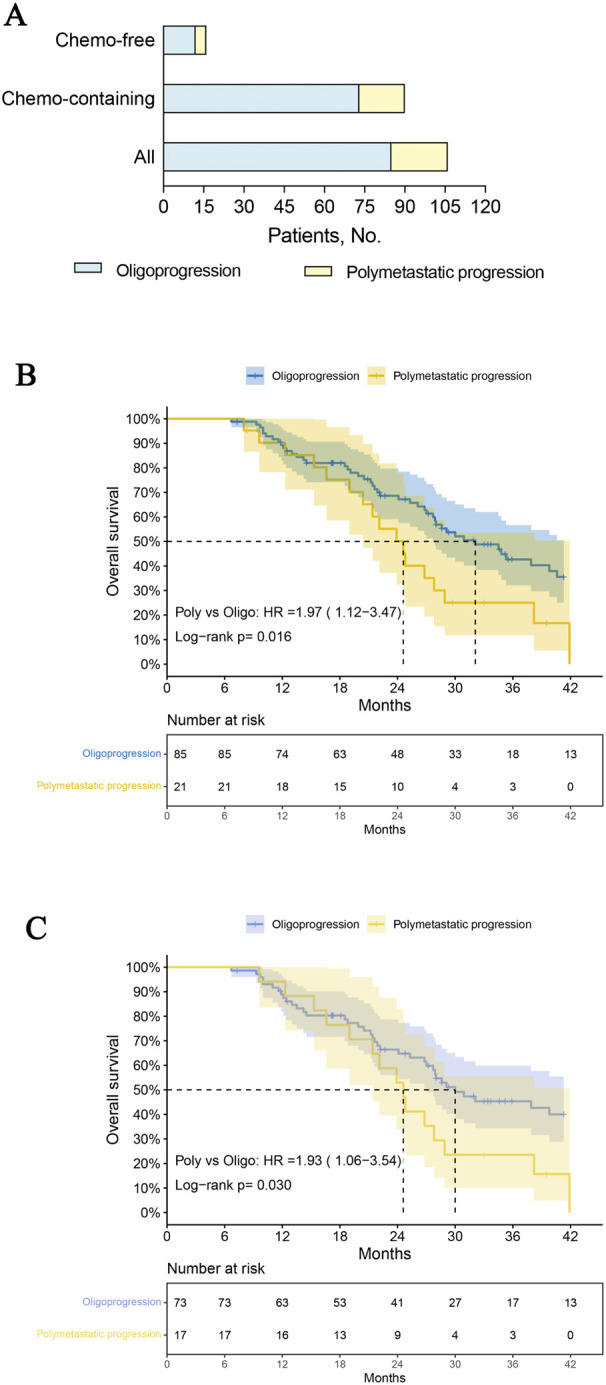
Progression patterns after acquired resistance (AR). Panel **(A)** shows the distribution of oligoprogression and polymetastatic progression across cohorts. Panel **(B)** shows Kaplan–Meier overall survival curves for patients with AR according to progression pattern in the overall cohort. Panel **(C)** shows Kaplan–Meier overall survival curves for patients with AR according to progression pattern in the chemotherapy-containing cohort. AR, acquired resistance; HR, hazard ratio.

At the time of acquired resistance, oligoprogression was the most common progression pattern in the overall AR cohort. Of the 106 patients, 85 (80.2%) developed oligoprogression, whereas 21 (19.8%) developed polymetastatic progression (**Figure 3A**). A similar distribution was observed in the chemotherapy-containing cohort, in which 73 of 90 patients (81.1%) had oligoprogression and 17 (18.9%) had polymetastatic progression. Among the 16 patients treated with non-chemotherapy-containing regimens, 12 (75.0%) developed oligoprogression and 4 (25.0%) had polymetastatic progression.

Within the overall cohort, OS was significantly shorter in patients with polymetastatic progression than in those with oligoprogression (HR = 1.97, 95% CI 1.12–3.47; p = 0.016; **Figure 3B**). We next examined this association among patients receiving chemotherapy-containing regimens. In this cohort, OS also remained poorer in patients with polymetastatic progression than in those with oligoprogression (HR = 1.93, 95% CI 1.06–3.54; p = 0.030; **Figure 3C**).

Cox regression analyses were further used to assess factors influencing OS after acquired resistance. Among the variables examined, only progression pattern showed a significant association with OS in univariable analysis (HR = 0.51, 95% CI 0.29–0.89; p = 0.018). This finding persisted in the multivariable model, where oligoprogression emerged as an independent favorable prognostic factor for OS (HR = 0.45, 95% CI 0.25–0.82; p = 0.009). Additional results are available in the **Supplementary Material**.

### Site of AR

**Figure 4:**
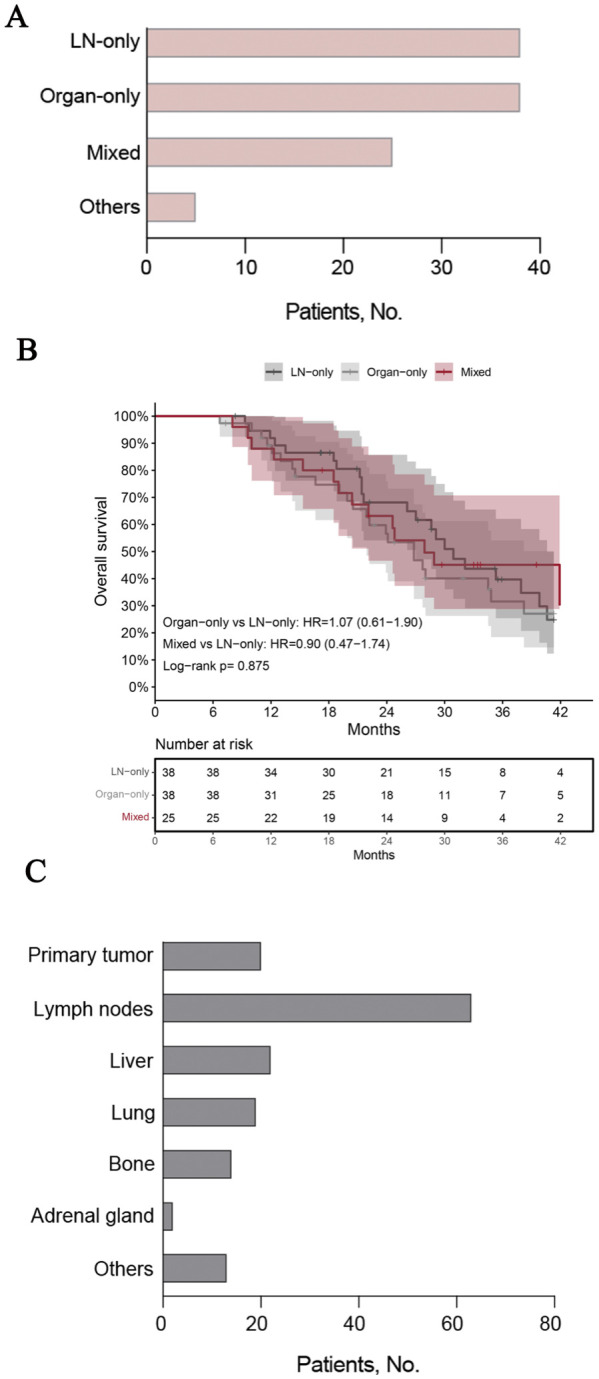
Sites of acquired resistance (AR). Panel **(A)** shows the distribution of lymph node-only, organ-only, mixed, and other sites of AR. Panel **(B)** shows Kaplan–Meier overall survival curves for patients with AR according to site category. Panel **(C)** shows the distribution of individual progression sites. AR, acquired resistance; HR, hazard ratio; LN, lymph node.

We next assessed progression sites at acquired resistance. LN-only and organ-only progression were the most common patterns, each occurring in 38 patients, followed by mixed progression in 25 patients and other patterns in 5 patients (**Figure 4A**). OS did not differ significantly among patients with LN-only, organ-only, and mixed progression (p = 0.875; **Figure 4B**). Compared with LN-only progression, neither organ-only progression (HR = 1.07, 95% CI 0.61–1.90) nor mixed progression (HR = 0.90, 95% CI 0.47–1.74) was associated with a significant difference in risk. At the individual-site level, lymph nodes were the most frequent sites of progression, followed by liver, primary tumor, lung, and bone (**Figure 4C**).

### Management after AR

Post-AR treatment information was available for 84 patients. Systemic therapy predominated after AR, being used in 82 patients; local treatment was recorded in 10 patients. Within the systemic-treatment population, ICI-containing regimens were the most common approach (66/82, 80.5%). The remaining patients received non-ICI systemic strategies, including chemotherapy, targeted therapy, or novel-agent–based therapy.

## Discussion

We performed a multicenter retrospective analysis to characterize the clinical presentation of acquired resistance in metastatic or unresectable locally advanced ESCC after initial clinical benefit from first-line ICI-based therapy, with particular focus on progression patterns and sites. We found that oligoprogression was the predominant pattern of acquired resistance, whereas polymetastatic progression was associated with significantly worse overall survival. By contrast, progression-site categories did not significantly stratify survival.

Immunotherapy resistance is commonly classified as primary or acquired (9, 14). Primary resistance reflects failure to achieve meaningful tumor control after treatment initiation, whereas acquired resistance is characterized by progression after an initial period of response or sustained disease control (14). Our study focused on acquired resistance after initial clinical benefit. Current evidence suggests that acquired resistance may arise through multiple, potentially overlapping mechanisms, including impaired antigen presentation, disruption of interferon-γ signaling, selection of resistant subclones, activation of alternative inhibitory pathways, and remodeling of the tumor microenvironment (9, 17–20). These mechanisms may also be shaped by the specific treatment backbone used with immune checkpoint blockade (21). ICI monotherapy primarily imposes immune-mediated selection pressure on tumor clones and microenvironments capable of escaping reinvigorated antitumor T-cell responses. By contrast, chemotherapy-containing regimens may additionally influence antigen release, immunogenic cell death, myeloid-cell composition, lymphocyte reserve, and immune-cell infiltration (22–24). Targeted agents, particularly antiangiogenic therapies, may further modify vascular normalization, hypoxia, immune-cell trafficking, and stromal–immune interactions (25). Therefore, different first-line ICI-based regimens could theoretically contribute to distinct patterns of resistance evolution and may partly influence whether AR emerges as limited-site progression or as more disseminated disease. However, our cohort was not designed to test regimen-specific resistance mechanisms. The small number of patients receiving non-chemotherapy-containing regimens, together with the lack of paired tissue, blood-based biomarker, and spatial immune-profiling data at baseline and at AR, precluded robust comparisons of immune-editing pressure across treatment regimens. Future prospective studies should integrate regimen-stratified clinical analyses with longitudinal biospecimen collection, next-generation sequencing, multiplex immunohistochemistry, spatial profiling, and immune-cell characterization to clarify how different ICI-based combinations shape resistance evolution and AR progression patterns (26). These considerations support the view that acquired resistance is unlikely to represent a biologically uniform process, and that different resistance trajectories may lead to distinct patterns of relapse. In our cohort, oligoprogression and polymetastatic progression were associated with different survival outcomes, supporting the clinical relevance of this classification. A plausible explanation is that oligoprogression reflects resistance confined to a limited number of anatomical sites, whereas polymetastatic progression indicates more widespread treatment failure. This may also explain why progression pattern, rather than the anatomical category of progression sites, showed greater prognostic value after acquired resistance.

Across tumor types, acquired resistance often manifests initially as limited rather than widespread progression. In the Peking University gastrointestinal cancer cohort, oligoprogression accounted for 70.5% of acquired resistance events, lymph nodes were the most common progression sites (58.4%), and oligoprogression was associated with longer overall survival than polymetastatic progression (38.5 vs 14.0; HR = 0.37, 95% CI 0.18–0.74; p < 0.001) (12). In NSCLC, Gettinger et al. reported recurrence confined to one or two sites in 88% of patients, with lymph nodes among the most frequent sites of failure, while Xu et al. identified oligoprogression as the dominant post-resistance pattern (55.3%) and associated it with better survival than systemic progression (25.8 vs 19.1; p = 0.003) (10, 27). Similarly, a recent study in patients with metastatic esophageal cancer following failure of first-line immunotherapy showed that oligoprogression was slightly more frequent than polyprogression (53.7% vs 46.3%), and that lymph nodes were again the predominant sites of progression (28).

Our definition of oligoprogression should be interpreted within the specific context of acquired resistance after immune checkpoint inhibitor therapy. Rather than applying a formal oligometastatic-disease framework, which commonly incorporates the absolute number of metastatic lesions and the feasibility of treating all lesions with local therapy, we classified progression according to the number of involved anatomical sites at the time of AR (29). This approach is consistent with prior studies of acquired resistance or post-immunotherapy progression patterns, in which limited progression has frequently been characterized by involvement of only a small number of disease or failure sites (10, 12, 16, 27). Nevertheless, lesion-level disease burden remains clinically relevant. Multiple progressing lesions within the same organ may not be biologically equivalent to a solitary progressing lesion, even if both are counted as one involved site. Therefore, our findings should be viewed as describing the anatomical extent of AR, and future studies should refine oligoprogression definitions by integrating both site-level and lesion-level information.

These findings suggest that oligoprogression and polymetastatic progression may reflect different degrees of immune escape. Under treatment pressure, tumors are unlikely to evade immune control in a uniform manner across all lesions. In some patients, resistant disease may remain restricted to a limited number of sites and present clinically as oligoprogression; in others, resistance may become more disseminated. Mechanistically, this may reflect impaired antigen presentation, diminished responsiveness to interferon-γ signaling, activation of alternative inhibitory pathways such as LAG-3 or TIGIT, or a shift towards local immune suppression characterized by the accumulation of regulatory T cells and myeloid-derived suppressor cells, together with terminally exhausted CD8+ T-cell states (9, 17–20, 30, 31). Once resistant clones are no longer confined to isolated sites, progression may be more likely to assume a polymetastatic pattern.

By contrast, progression-site categories did not significantly stratify overall survival in our cohort, possibly because site category captures where progression occurs, but not how extensively resistant disease has spread. Lymph node-only, organ-only, and mixed progression differ anatomically, but do not necessarily reflect the overall burden or biological aggressiveness of post-AR disease. Organ-only progression may still represent limited resistant disease, whereas lymph node-only progression may already indicate more extensive regional dissemination. Accordingly, anatomical category alone may be insufficient to capture the clinical heterogeneity of acquired resistance. Our data support progression pattern as a more informative prognostic marker than site category in ESCC after acquired resistance.

At present, clinical studies on acquired resistance have mainly involved mixed gastrointestinal cohorts, melanoma, NSCLC, and metastatic esophageal cancer after immunotherapy failure. Data focused specifically on ESCC remain limited, particularly for patients who developed acquired resistance after initial clinical benefit from first-line ICI-based therapy. Against this background, our study adds evidence focused on ESCC by examining progression patterns, progression sites, and their associations with survival after acquired resistance to first-line ICI-based therapy.

The predominance of oligoprogression also has potential management implications. Current clinical practice guidelines for esophageal cancer emphasize systemic therapy as the main treatment backbone for recurrent or metastatic disease, while treatment decisions should be individualized according to disease extent, symptoms, performance status, prior therapy, and multidisciplinary assessment (32). In this context, oligoprogression after initial benefit from ICI-based therapy may represent a clinically relevant state in which resistant disease is confined to a limited number of sites, while other disease sites may remain controlled by ongoing systemic treatment. For selected patients, local treatment to progressing lesions, such as radiotherapy, surgery, or other ablative approaches, combined with continuation or optimization of systemic therapy, may therefore be considered after multidisciplinary evaluation.

Evidence from ICI-treated solid tumors also supports this approach, as local ablative radiotherapy for oligoprogression has been associated with prolonged disease control and delayed systemic treatment change (33, 34). In recurrent or metastatic esophageal cancer, retrospective evidence also suggests that adding radiotherapy to chemoimmunotherapy may improve survival, although prospective validation remains necessary (35). Accordingly, the distinction between oligoprogression and polymetastatic progression in our cohort should not be interpreted solely as a prognostic classification. It may also help identify patients who warrant careful reassessment for local control strategies, continuation or modification of systemic therapy, and closer post-AR surveillance. Therefore, although oligoprogression may help identify patients for whom local control strategies deserve consideration, our data do not establish the optimal post-AR treatment approach. The value of post-AR management should not be judged by survival alone. In advanced ESCC, treatment after resistance may also affect dysphagia, symptom burden, nutritional status, functional status, treatment-related toxicity, and overall quality of life. Because quality-of-life and patient-reported outcome data were not systematically collected in this retrospective cohort, these aspects could not be evaluated. Future prospective studies should incorporate standardized patient-reported outcomes alongside survival endpoints to provide a more complete assessment of post-AR treatment benefit (36).

This study has several limitations. First, its retrospective design introduces the possibility of selection bias and missing clinical information. Second, the cohort was modest in size, partly because the study population was narrowly defined and restricted to ESCC patients who developed acquired resistance after initial clinical benefit from first-line ICI-based therapy. Third, post-AR treatment was heterogeneous and may have affected survival outcomes. Moreover, quality-of-life and patient-reported outcome data were not systematically available, limiting the assessment of symptoms, functional status, treatment tolerability, and overall quality of life after AR. Fourth, progression patterns were defined according to the number of involved anatomical sites, and lesion-level burden within each site was not systematically captured. This may have obscured heterogeneity within the oligoprogression group, particularly when multiple lesions progressed within the same organ. Future studies incorporating both site-level and lesion-level information may provide a more refined framework for defining oligoprogression after immune checkpoint inhibitor therapy and for improving post-AR risk stratification. Finally, molecular and biomarker data were not systematically available to support further biological interpretation. PD-L1 expression and TMB results were available for only a small subset of patients. This was largely because biomarker testing was not uniformly performed in this real-world retrospective cohort, and retrospective extraction was constrained by incomplete medical records, variable testing platforms, and limited availability of archival tumor tissue. Therefore, analyses examining the associations of PD-L1 expression or TMB with AR patterns would have been underpowered and susceptible to selection bias. Given the established clinical relevance of PD-L1 expression in first-line immunotherapy for advanced ESCC and the emerging role of TMB-related metrics in predicting immunotherapy outcomes, future prospective studies should incorporate standardized PD-L1 assessment and sequencing-based TMB evaluation to clarify their relationship with AR patterns and post-AR survival (2, 37). Longitudinal biomarker approaches, including ctDNA profiling where feasible, may further support biology-driven surveillance and post-AR risk stratification in ESCC (38). HPV status was also not routinely assessed in this cohort. Because the prognostic significance of HPV infection in ESCC remains incompletely defined, future studies should further determine whether viral status contributes to biological heterogeneity after acquired resistance (39).

Overall, our results indicate that progression pattern after acquired resistance carries prognostic relevance in ESCC. Oligoprogression and polymetastatic progression were associated with different survival outcomes, whereas progression-site category was not clearly prognostic in this cohort. This distinction may be useful for post-AR risk stratification in clinical practice. However, management after AR remains heterogeneous, and the optimal treatment approach is still uncertain.

## Conclusions

In conclusion, oligoprogression represented the predominant pattern of acquired resistance in advanced ESCC after initial clinical benefit from first-line ICI-based therapy, whereas polymetastatic progression was associated with worse survival and remained independently prognostic. By contrast, progression-site categories did not significantly stratify survival. These findings suggest that progression extent may be more informative than progression-site category for prognostic assessment after acquired resistance. The optimal management strategy after AR in ESCC remains to be defined.

## Data Availability

The data used in this study are not publicly available because they contain patient-level clinical information. However, de-identified data may be obtained from the corresponding author upon reasonable request and in accordance with applicable ethical and institutional requirements.
