# Supplementary material for: Acquired resistance to first-line immune checkpoint inhibitors in esophageal squamous cell carcinoma: progression patterns and prognostic implications
Source: Front Immunol. 2026 Jul 23;17:1906395. doi: 10.3389/fimmu.2026.1906395 (PMC13443299; doi:10.3389/fimmu.2026.1906395)
Supplement: Supplementary file 1 [file DataSheet1.pdf]

Supplementary Material

Supplementary Table S1. Univariate Cox proportional hazards analysis of overall survival (OS)

Supplementary Figure S1. Forest plot of multivariable Cox proportional hazards analysis for overall survival (OS)

Supplementary Table S2. Key baseline characteristics according to study center

Table S1. Univariate Cox regression analysis for overall survival

Variables	Category	HR (95% CI)	P value
Age (≥65 years)	≥65	1.09 (0.66–1.78)	0.741
Sex	Male	1.14 (0.63–2.08)	0.660
Smoking status	Yes	1.25 (0.75–2.07)	0.388
Alcohol consumption	Yes	1.47 (0.83–2.60)	0.186
Tumor location	Middle	1.82 (0.76–4.33)	0.176
	Lower	1.55 (0.62–3.83)	0.348
Prior surgery	Yes	0.87 (0.52–1.48)	0.611
Distant metastasis (M1)	Yes	1.00 (0.55–1.82)	0.989
Lymph node metastasis	Yes	0.74 (0.38–1.47)	0.393
ICI plus chemotherapy	Yes	0.93 (0.46–1.89)	0.842
Progression pattern	Oligoprogression	0.51 (0.29–0.89)	0.018

Abbreviations: HR, hazard ratio; CI, confidence interval; ICI, immune checkpoint inhibitor.

Reference categories: age <65 years, female sex, non-smoking, non-alcohol consumption, upper tumor location, no prior surgery, absence of distant metastasis, absence of lymph node metastasis, no ICI plus chemotherapy, and systemic progression.

Methods: Hazard ratios (HRs) with 95% confidence intervals (CIs) were estimated using univariate Cox proportional hazards regression models. All variables were analyzed as categorical variables.

Notes: P values were derived from two-sided tests, and statistical significance was defined as $P < 0.05$.

Figure S1. Forest plot of multivariable Cox proportional hazards analysis for OS

Variable	HR (95% CI)	P value
**Progression pattern**		
Poly	Reference	
Oligo	0.45 (0.25, 0.82)	0.009
**Age**		
<65 year	Reference	
≥65 year	1.01 (0.60, 1.69)	0.975
**Chemotherapy-containing therapy**		
No	Reference	
Yes	0.95 (0.46, 1.96)	0.891
**Metastatic disease**		
No	Reference	
Yes	1.04(0.55, 1.97)	0.901
**Primary tumor location**		
Upper	Reference	
Middle	1.88 (0.78, 4.51)	0.160
Lower	1.30(0.51, 3.34)	0.585
**Prior surgery**		
No	Reference	
Yes	0.82 (0.47, 1.43)	0.484

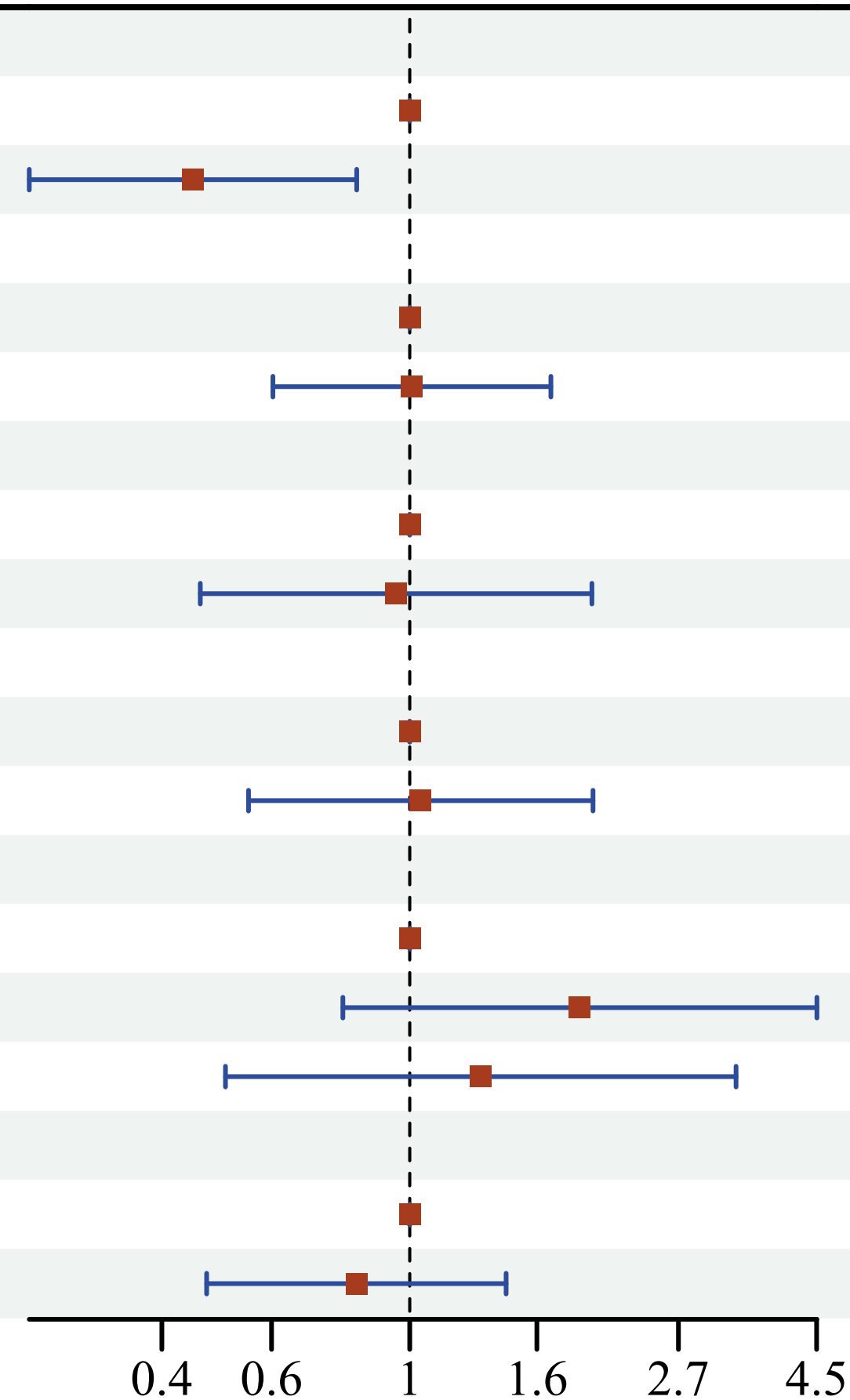

Supplementary Table S1. Key baseline characteristics according to study center

Characteristic	Overall cohort, n = 106		P value
	The First Affiliated Hospital of Zhengzhou University, n = 96	Anyang Cancer Hospital, n = 10	
**Age, n (%)**			0.753
<65 years	43 (44.8)	5 (50.0)	
≥65 years	53 (55.2)	5 (50.0)	
**Sex, n (%)**			0.229
Male	76 (79.2)	6 (60.0)	
Female	20 (20.8)	4 (40.0)	
**Disease status, n (%)**			1.000
Locally advanced unresectable	20 (20.8)	2 (20.0)	
Metastatic	76 (79.2)	8 (80.0)	
**Primary tumor location, n (%)**			0.620
Upper	11 (11.5)	0 (0.0)	
Middle	57 (59.4)	6 (60.0)	
Lower	28 (29.2)	4 (40.0)	
Baseline lymph node involvement, n (%)	84 (87.5)	8 (80.0)	0.618
Baseline liver involvement, n (%)	14 (14.6)	3 (30.0)	0.200
Baseline lung involvement, n (%)	25 (26.0)	2 (20.0)	1.000
Baseline other distant organ involvement, n (%)	15 (15.6)	2 (20.0)	0.661
Previous surgery, n (%)	25 (26.0)	6 (60.0)	0.060
Previous radiotherapy, n (%)	8 (8.3)	0 (0.0)	1.000
**Treatment category, n (%)**			1.000
Chemotherapy-containing	81 (84.4)	9 (90.0)	
Non-chemotherapy-containing	15 (15.6)	1 (10.0)	
**Initial clinical benefit, n (%)**			0.086
PR	57 (59.4)	9 (90.0)	
SD ≥6 months	39 (40.6)	1 (10.0)	

Data are presented as n (%). P values were calculated using Fisher's exact test. Because the number of eligible patients from Anyang Cancer Hospital was limited, center-level comparisons were considered descriptive. PR, partial response; SD, stable disease.
